# Finding quasi-modules of human and viral miRNAs: a case study of human cytomegalovirus (HCMV)

**DOI:** 10.1186/1471-2105-13-322

**Published:** 2012-12-03

**Authors:** Isana Veksler-Lublinsky, Yonat Shemer-Avni, Eti Meiri, Zvi Bentwich, Klara Kedem, Michal Ziv-Ukelson

**Affiliations:** 1 Department of Computer Science, Ben-Gurion University, Beer-Sheva 84105, Israel; 2 Virology and Developmental Genetics/Health Sciences, Ben-Gurion University; 3, Rosetta Genomics Ltd., 10 Plaut St., Rehovot, 76706, Israel

## Abstract

**Background:**

MicroRNAs (miRNAs) are important regulators of gene expression encoded by a variety of organisms, including viruses. Although the function of most of the viral miRNAs is currently unknown, there is evidence that both viral and host miRNAs contribute to the interactions between viruses and their hosts. miRNAs constitute a complex combinatorial network, where one miRNA may target many genes and one gene may be targeted by multiple miRNAs. In particular, viral and host miRNAs may also have mutual target genes. Based on published evidence linking viral and host miRNAs there are three modes of mutual regulation: competing, cooperating, and compensating modes.

**Results:**

In this paper we explore the compensating mode of mutual regulation upon Human Cytomegalovirus (HCMV) infection, when host miRNAs are down regulated and viral miRNAs compensate by mimicking their function. To achieve this, we develop a new algorithm which finds groups, called *quasi-modules*, of viral and host miRNAs and their mutual target genes, and use a new host miRNA expression data for HCMV-infected and uninfected cells. For two of the reported quasi-modules, supporting evidence from biological and medical literature is provided.

**Conclusions:**

The modules found by our method may advance the understanding of the role of miRNAs in host-viral interactions, and the genes in these modules may serve as candidates for further experimental validation.

## Background

MicroRNAs (miRNAs) are an abundant class of small noncoding RNAs (20–24 nts) that regulate gene expression by usually binding 3’ UTRs of mRNA target transcripts. They serve as major regulators of many biological processes such as development, differentiation, growth and apoptosis. The human genome encodes over 1400 miRNAs [1], and miRNAs are also encoded by viruses, mainly herpes-viruses [2].

The targets for the majority of viral miRNAs are currently unknown, however, recent reports show various roles for them in blocking apoptosis, in immune evasion and in regulation of viral replication through targeting both host and viral genes [3-5]. In addition to expressing their own miRNAs, infections with some viruses can result in changes in the expression of host miRNAs. These changes can be the outcome of host response to the infection, and/or changes induced by the virus, to its own benefit [6,7].

The participation of miRNAs in host-viral interactions makes them attractive targets for antiviral therapy. Thus, there is a motivation to identify the target genes of both host and viral miRNAs. Over the years, several computational algorithms and tools for target prediction have been developed (for a review see [8-10]). However, these tools are noisy and predict an excess of targets for each miRNA, with a very high false-positive rate, which stands in the way of experimental wet-lab validation. This limitation is due to the fact that miRNAs are very short and their interaction with target genes is not very specific. To overcome this limitation, we predict combinatorial miRNA-target interactions rather than single interactions. Additional context is added to the prediction by considering different modes of mutual regulation by host and viral miRNAs. We seek groups of viral and human miRNAs and their common target genes (*modules*). To achieve this, we combine target prediction results with additional information sources (e.g., GO/KEGG categories and miRNA expression data). This approach may narrow the list of target genes to more reliable candidates.

Previous studies show that one miRNA may have several target genes and that one mRNA can be targeted by multiple miRNAs [11-13], in particular viral and host miRNAs may also have mutual targets. Based on the literature, there are three modes of mutual regulation by host and viral miRNAs: a competing mode, a cooperating mode and a compensating mode. First, human and viral miRNAs may *compete* for target sites (if the target sites are overlapping). Second, they could *cooperate* to enhance the down regulation of their mutual targets. Third, through alternation of viral/host miRNA expressions, viral and host miRNAs could *compensate* each other’s action in the target regulation task. Below we supply evidence from literature for these modes and bioinformatically analyze mode 3.

As evidence to the first mode, Nachmani et al. [14] report that hcmv-miR-UL112-1 and hsa-miR-373, which have overlapping sites on the MICB mRNA, showed competitive mutual gene regulation. The authors note that the strategy in which the viral miRNAs target a binding site that is already in use by host miRNAs, makes it extremely difficult for the host to escape viral regulation by mutating the relevant binding site.

The second mode of cooperativity between viral and host miRNAs in host gene regulation, was proposed and bioinformatically explored in our previous work [15]. In that previous study, the hypothesis was that viral miRNAs that share targets with human miRNAs contribute to increasing the translational repression and tightening the regulation which already existed at a modest level in the cell (by the host regulation machinery). Our system predicted groups of human and EBV miRNAs that may mutually regulate human mRNAs enriched in several biological processes related to EBV. Biological support to this mode was given by Nachmani et al. [14] showing that hcmv-miR-UL112-1 and hsa-miR-376, which have distinct target sites on MICB mRNA, cooperate within infected cells to down-regulate MICB. Furthermore, a recent study which used the HITS-CLIP method on EBV-infected cells, reported that about 1500 human genes are targeted by both EBV and human miRNAs during latency, via distinct binding sites [16].

As for mode 3, Skalsky et al. [17,18] reported that Kaposi’s sarcoma-associated herpesvirus (KSHV) miR-K12-11 is an ortholog of cellular miR-155. These two miRNAs are identical along their 5’ terminal 8 nts, including the entire seed region, and it was determined experimentally that both miRNAs share several target genes [17]. Furthermore, O’Hara *et al.*[19] reported that miR-155 was down-regulated in Kaposi-sarcoma cancer, thus the authors suggested that its viral ortholog could compensate for some of its functions. Given that there are only a few sequence mimicry examples in the literature (reviewed in [20]), we decided to investigate deeper the compensation mode of regulation, where viral miRNAs mimic the function of host miRNAs. We seek bioinformatically modules of host and viral miRNAs and their common target genes, where the host miRNAs are down regulated upon viral infection, and viral miRNAs compensate for this.

Previous studies have developed methods for finding modules consisting of miRNAs and genes of the same species. We refer to some of them [21-23], in our paper [15]. Recently, Xu et al., [24] identified miRNA pairs, in order to analyze their functions, based on GO and protein–protein interactions. Kim et al. [25] used a layered hypernetwork (LHNs) model to find functional miRNA-mRNA regulatory modules from expression profiles. Peng et al. [26] presented a method which combines the inverse expression relationship between miRNAs and mRNAs with target prediction to find miRNA-mRNA modules, and used it to infer human miRNA-mRNA modules associated with HCV infection.

The common way to find the desired modules in the methods above is by first representing the multiple relations between miRNAs and target genes by a bipartite graph, where an edge indicates that the miRNA targets the gene. Next, by finding bi-cliques in the graph, which represent the miRNA–mRNA modules. In general, finding bi-cliques in bi-partite graphs can be formulated as a bi-clustering problem (reviewed in [27]), which is known to be NP-complete. Therefore, most of the methods that address bi-clustering are based on heuristic approaches, which may miss good solutions. Instead, we developed in our previous work [15] an enumeration method which does not miss any of the possible modules. Furthermore, to enforce modules to consist of both viral and host miRNAs each set of miRNAs (host, viral) has to be clustered separately (bi-targeting method).

**Figure 1:**
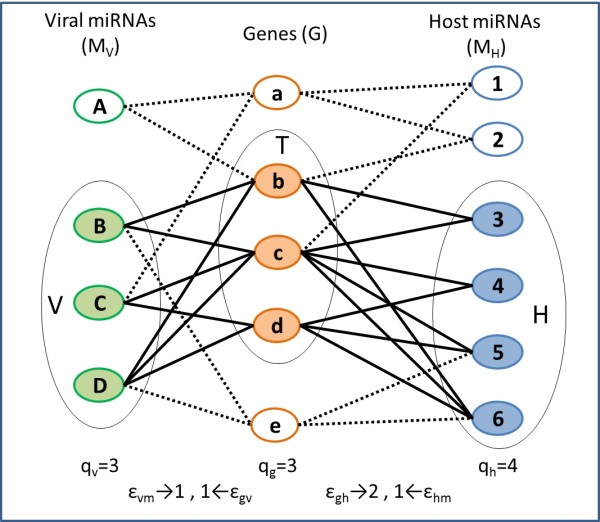
**The two–sided bipartite graph.** There is an edge between an miRNA and a gene if the gene is targeted by that miRNA. The quorum and error thresholds are listed below the graph. A quasi-module in this graph consists e.g. of the shaded circles (the sets *T*, *H* and *V* and the module edges are the thick lines). The full set of quasi-modules for this graph is found in Figure 2.

In that work we built a two sided miRNA-mRNA-miRNA input graph (for example see Figure 1) using the target prediction information, and found in the graph maximal two-sided complete bi-cliques that followed quorum (minimal number) constraints on the number of human and viral miRNAs in the module. The algorithm was applied for searching for EBV-human modules. The disadvantage of this method was that the strong requirement that the sought modules be complete bi-cliques (i.e. modules in which all miRNAs target all genes) constrained the results and yielded very few, small modules. Furthermore, we expect that in some modules not all miRNAs would target all genes. Some miRNA-target relationships could be missing due to either false negatives in the target prediction or because the natural co-regulation process does not necessarily require that all miRNAs in the module target all the genes.

**Figure 2:**
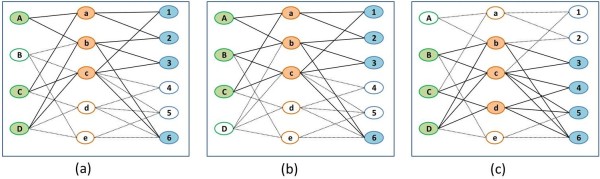
**The resulting quasi-modules found in the data in Figure**1**.** The three modules, denoted as (**a**), (**b**) and (**c**), consist of the shaded circles and the thick lines.

We add flexibility to the modules, by devising a new *quasi* bi-targeting algorithm which computes quasi bi-cliques of human and viral miRNAs and their target genes. Mining of quasi bi-cliques has been previously successfully applied in fields like the stock market and protein networks [28]. Our algorithm combines and extends approaches from [15] and [28] to yield a new method to compute the *quasi-modules*. A quasi-module is represented by a subgraph of the input graph consisting of three disjoint sets of vertices (see Figure 1): human miRNAs *H*, viral miRNAs *V* and target genes *T*, and their corresponding edges. Given the error tolerances *ϵ*_*hm*_, *ϵ*_*vm*_, *ϵ*_*gh*_ and *ϵ*_*gv*_, the module must comply with the following criteria: every miRNA in *H* and *V* has to connect with all but *ϵ*_*hm*_ and *ϵ*_*vm*_genes in *T*, respectively. Every gene in *T* has to connect with all but *ϵ*_*gh*_ and *ϵ*_*gv*_miRNAs in *H* and *V*, respectively. In Figure 1, the shaded circles form a quasi-module, where *T*={*bcd*},*H*={3,4,5,6},*V*={*BCD*}, and *ϵ*_*hm*_=*ϵ*_*vm*_=*ϵ*_*gv*_=1 and *ϵ*_*gh*_=2. In this quasi-module, every human and viral miRNA can be disconnected with up to one gene (*ϵ*_*hm*_, *ϵ*_*vm*_), and every gene can be disconnected with up to two human miRNAs (*ϵ*_*gh*_) and up to one viral miRNA (*ϵ*_*gv*_). In total, three quasi-modules that comply with the above criteria are found in the graph (see Figure 2).

In addition, we supply new expression data of human miRNAs in Human cytomegalovirus (HCMV) infected vs un-infected cells. We extract from this data human miRNAs that are significantly down-regulated upon infection, and use our new relaxed bi-targeting algorithm to study the compensating regulation of miRNAs in HCMV infection. We perform the search for modules on all KEGG pathways and the significant modules are picked by a sampling procedure. We validate two of the modules, found in pathways that are related to HCMV biology, by surveying information from the biological and biomedical literature. The rest of the significant modules identified in our study can be found at http://www.cs.bgu.ac.il/∼vaksler/QuasiBiTargeting.html.

Genes that are included in our modules, may serve as better candidates for experimental target validation, since they are predicted to be targeted by multiple viral and human miRNAs, whose expression in uninfected vs. infected cells is in accordance with mode 3. We believe that our results may contribute to a wider understanding of viral-induced diseases and the role that miRNAs plays in them.

## Methods

### Datasets

The human and viral mature miRNA sequences, were downloaded from the miRNA registry [1]. Additional sequences of HCMV miRNAs that were reported recently by Stark et al. [29] and Meshesha et al. [30], were also taken into account. The full set of HCMV miRNAs appears in Table 1. 

**Table 1:** Viral miRNAs in the study, divided into two groups

	**hcmv-miR-**	**No. of targets**
	US25-1-3p	49
	US5-2-3p	70
	UL36-5p	92
	US5-1-3p	97
	UL22A-5p	176
	UL112-3p	186
A	US25-1-5p	212
	US33-3p	278
	US25-2-3p	310
	UL22A-3p	310
	US33-5p	320
	US25-2-5p	352
	UL36-3p	861
	US5-2-5p	71
	US22-3p	102
	US4-3p	271
	UL112-5p	295
B	US4-5p-shift5^∗^	297
	US29-3p	314
	US29-5p^∗∗^	339
	US22-5p	482

A) miRNAs from miRBase, Release 18 [1]; (B) additional miRNAs recently found by Deep Sequencing [29,30]. Each group is sorted by the number of targets. ^∗^The sequence of miR-US4-5p that was detected by deep sequencing in [29,30] was shifted by 5 bp at the 5^′^end from the miRBase sequence, we included the shifted sequence in the datasets. ^∗∗^Denoted as US33a in [29]. In addition, mature miRNAs from miR-UL70 were not detected by the deep-sequencing, thus they were not included in the analysis.

In the human miRNA dataset we included miRNAs whose expression was down-regulated upon HCMV infection according to at least one of the following sources: our new expression data, data from [31] and data from [32](see below). The full set of human miRNAs appears in Table 2. 

**Table 2:** Human miRNAs used in the study

	**hsa-**	**No. of targets**
	miR-99a	71
	miR-181a*	85
	miR-125b-1*	137
	miR-424*	151
	miR-155	153
	miR-21*	177
	miR-181a	245
	miR-29b	273
	miR-221	302
	miR-181b	308
	miR-222*	314
A	miR-886-3p	334
	miR-221*	378
	miR-199a-3p	408
	miR-484	408
	miR-708	446
	miR-214*	463
	miR-503	478
	miR-320a	493
	miR-214	624
	miR-29b-1*	633
	miR-34a	840
	miR-100	68
	miR-21	109
B	miR-223	118
	miR-101	196
	miR-199a-5p	256
	miR-222	340

Human miRNAs used in the study, that were shown to be down-regulated upon HCMV infection in at least one of the three expression data sources (data supplied in this study, by Wang et al. [31] and by Santhakumar et al. [32]). A) miRNAs that were classified as down regulated in the miRNA expression data supplied in this study (see Methods). B) Additional miRNAs that were classified as down regulated in the studies of Wang *et al*[31] and Santhakumar at el [32]. Each group is sorted by the number of targets.

The set of human 3’UTR sequences was extracted from the Ensembl’s Biomart database (Ensembl 53) [33]. The KEGG pathway lists of human genes were downloaded from [34]. The set of 3’UTRs was filtered to contain 3’ UTRs that belong to at least one pathway in the KEGG database. This resulted in 10925 sequences (different transcripts) from 5351 genes. In our study we used 207 KEGG pathways which contained 10-300 genes.

### Human miRNAs expression data

#### Cell cultures and RNA extraction

Human foreskin fibroblast (HFF) cells were grown in DMEM medium supplemented with 10% fetal calf serum (FCS), 1% L-glutamine and 1% penicillin/streptomycin (all reagents were supplied by Biological Industries, Beit Haemmek, Israel). Viral infection of HFF by HCMV (an isolate from clinical sample) was done at multiplicity of infection (MOI) 2. Three days after infection infected and mock infected HFF cells were harvested, and total RNA was isolated using EZ-RNA II kit (Biological Industries, Beit Haemmek, Israel) according to the manufacturer instruction. This procedure was carried out twice, resulting in two sets of miRNA expression measurements in infected vs un-infected cells. This data is available in a Additional file 1.

#### miRNAs Microarray analysis

The RNA was reverse transcribe and cRNA labeled with either cyanine 3-CTP (Cy3-CTP) or cyanine 5-CTP (Cy5-CTP) were generated from each cDNA source using the Low-Input Linear Amplification Kit (Agilent technologies, Santa Clara, USA) according to the manufacturer’s protocol, except that synthesis was initiated at the *in vitro* transcription step using 1 *μ*g of cDNA as starting material. Hybridization to the chip, (MIRCHIPTM, custom made, Agilent Technologies, Santa Clara CA, USA) displaying 45-mer oligonucleotide probes complementary to all human miRNAs and HCMV that were printed in triplicate spots, was carried out in solutions that contained the indicated amount of each of labeled cRNA from either the control or the test samples prepared using the In situ Hybridization Reagent Kit (Agilent). Hybridized microarrays were scanned using the Agilent LP2 DNA Microarray Scanner at 10 *μ*m resolution. Microarray images were visually inspected for defects. To each sample external spotted controls were added for normalization between samples. The initial data analysis was carried out by Rosetta Genomics.

#### Extracting down-regulated human miRNAs

For each of the two sets of expression measurements we calculated the ratio of the expression of infected to un-infected cells, and chose those miRNAs where at least one of the ratios, as well as the average of the two ratios were below 0.6. We filtered out miRNAs whose expression in un-infected cell was below 500. The chosen miRNAs are listed in Table 2(A).

Two additional studies measured the effect of HCMV on host miRNAs [31,32]. Wang et al. [31] used miRNA microarrays to measure host miRNA expression in HCMV infected cells (MRC-5 cells with CMV Towne BAC) in different time points (6, 24, 48, and 96, or 120 h post infection). From this report we chose miRNAs that had significant down-regulation in their expression levels in at least one time point. In a recent report by Santhakumar et al. [32] it was shown that miR-199a/214 cluster (miR-199a-5p, miR-199a-3p, and miR-214) was down-regulated in HCMV-infected cells, thus we included these miRNAs in our dataset. The additional miRNAs are listed in Table 2(B).

### The target prediction method

We ran all-against-all target prediction between the miRNA and 3’UTR sets described above. The target prediction was carried out with our previously developed tool described in [15] with the following constraints for each duplex: seed location is 2-8; maximum GU pairs in the seed is 1 ; maximum GU pairs in the duplex is 4; maximal number of mismatches/gaps in the duplex is 8; maximal size of a bulge is 6; the duplex free energy should be less than -17 kcal/mol or the normalized free energy score (normalized by the energy score of the miRNA bound to its perfect complement) should be greater than 0.4.

### Quasi-Bi-Targeting (QBT) enumeration algorithm

In this section we describe our approach to module composition. We start by constructing a two-sided bipartite graph with three sets of vertices. These sets are human miRNAs, viral miRNAs and human genes (see, for example, Figure 1), where the miRNAs are chosen by changes in expression levels (expression data) and the genes are restricted to belong to certain biological processes (GO/KEGG). An edge in the graph between an miRNA and a gene, indicates that this miRNA is predicted to target this gene (information on target prediction is supplied by applying our software [15], see details above). Once the graph is built, we use an enumeration algorithm to find in it quasi-modules that are statistically enriched in the explored biological process.

The enumeration algorithm described here extends our previous approach [15] for finding modules of miRNAs and their target genes. In addition to supporting quorum constraints on the number of genes and human and viral miRNAs in a sought module, we now support also an error tolerance threshold on the connectivity of the module.

The QBT algorithm relies on some Lemmas provided in [28]. The algorithm supplied there deals with bipartite graphs and supports equal quorum and error constraints for both sides of the graph. We extend these lemmas to fit two-sided bipartite graphs and different quorum and error constraints for each side of the graph (see example in Figure 1). In what follows we provide a formal definition of the problem and a high-level overview of the algorithm. In the Appendix we supply formal details along with observations and proofs which assert the correctness of the algorithm, an example which demonstrates the application of the algorithm and the pseudo-code for the algorithm.

#### Formal definition of the problem

Let *G* be a set of genes, coming from a given GO/KEGG category *C*. Let *M*_*H*_ be a set of human miRNAs, and *M*_*V*_ a set of viral miRNAs. We denote by *q*_*h*_and *q*_*v*_the minimal number (quorum) of human and viral miRNAs, respectively, and by *q*_*g*_ the minimal number (quorum) of target genes. Let *ϵ*_*hm*_, *ϵ*_*vm*_, *ϵ*_*gh*_ and *ϵ*_*gv*_ denote error tolerance thresholds, and *p* denote a p-value threshold.

Our goal is to compute quasi-modules defined as follows.

##### Definition 1

**A legitimate quasi-module**<*H*,*V*,*T*> consists of human miRNAs *H*⊆*M*_*H*_, viral miRNAs *V*⊆*M*_*V*_and their target genes *T*⊆*G*and fulfills: **(1)**∀*mir*∈*H*,|*T*(*mir*)|≥|*T*|−*ϵ*_*hm*_, **(2)**∀*mir*∈*V*,|*T*(*mir*)|≥|*T*|−*ϵ*_*vm*_, and **(3)**∀*gene*∈*T*,|*H*(*gene*)|≥|*H*|−*ϵ*_*gh*_and |*V*(*gene*)|≥|*V*|−*ϵ*_*gv*_, where *T*(*mir*)⊆*T*are genes targeted by miRNA *mir*, *H*(*gene*)⊆*H*and *V*(*gene*)⊆*V*are the human and viral miRNAs that target *gene*, respectively.

Among the quasi-modules computed, we focus on finding those that comply with the quorum criteria, such that |*H*|≥*q*_*h*_, |*V*|≥*q*_*v*_ and |*T*|≥*q*_*g*_, and whose target-set *T* yields an enrichment p-value smaller than *p* in *C*.

#### The enumeration tree

**Figure 3:**
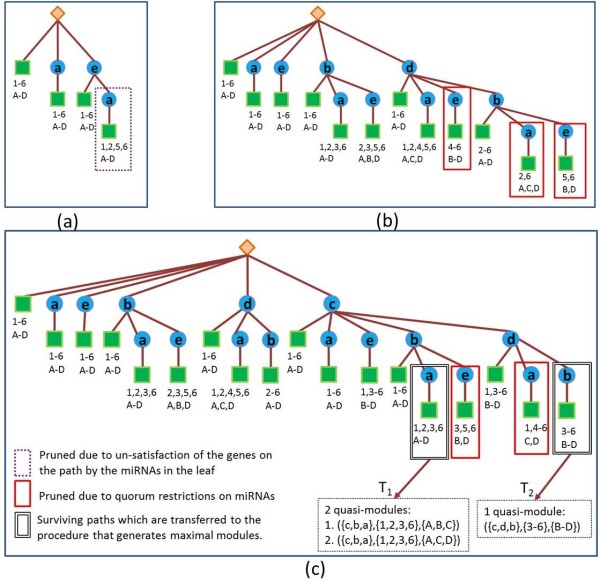
**The process of building the enumeration tree for the input graph in Figure**1**.** The rhombus represents the root of the tree. The circles represent the genes inserted into the tree. The squares represent the leaves which store two sets of miRNAs – human and viral (*H*(*T*) and *V*(*T*)). These miRNAs target at least |*T*|−*ϵ*_*hm*_and |*T*|−*ϵ*_*vm*_genes in *T* respectively, where *T* is the set of genes in the path from the leaf to the root. Viral miRNAs are labeled with uppercase letters and the human miRNAs are labeled with numbers. The rectangles (see legend) indicate paths that are pruned from the tree for one of two reasons: (1) paths where the miRNAs in the leaf break the quorum are in solid rectangles (in this example the required quorum is 3 for viral miRNAs and 4 for human miRNAs); (2) paths where at least one gene or a pair of genes do not satisfy the error constraint with respect to the miRNAs in the leaf are in dotted rectangles. Paths which reach the minimal number of genes, (in this example the number is 3), are forwarded to the next step of generating modules. The three sub-figures **(a)**, **(b)** and **(c)** show the insertion of genes *a*, *e*, *b*, *d* and *c*, into the tree, in this order.

Our enumeration algorithm dynamically constructs an enumeration tree of all possible modules (Figure 3). The enumeration tree consists of a root which is a dummy node, inner nodes which correspond to genes, and leaf nodes which contain a list of human and viral miRNAs. A path from the root to a leaf corresponds to a subgraph in the two-sided bipartite graph, consisting of all the genes appearing in internal nodes on this path and the miRNAs listed in the leaf. This subgraph contains one or more modules that comply with the quorum constraints and the error tolerant thresholds (according to Definition 1). Each such legitimate module consists of all the genes in the subgraph and a subset of the miRNAs (Figures 2 and 3).

#### High-level overview of the algorithm

The algorithm consists of two stages, where the first stage constructs the enumeration tree and the second stage extracts, for each path in the enumeration tree, the legitimate modules encoded by the corresponding subgraphs.

**Stage 1: Constructing the enumeration tree.** The tree is initialized to consist of a root node and one dummy leaf node, such that the leaf’s miRNA sets consist of all human and viral miRNAs in the dataset. Then genes are inserted to the tree one by one. Each inserted gene, *g*, is connected by an edge to the root and becomes a root of newly generated copies of all its preceding siblings in the tree including their corresponding subtrees. The miRNA sets in the leaves are computed for the new paths by updating the copied sets to comply with the addition of the new node. A path in the tree is pruned if one of the following conditions is violated, (i) the miRNAs in the leaf do not satisfy the quorum constraints, (ii) the error constraints for the genes on the path are not satisfied by the miRNAs in the leaf. (The fact that such pruning is safe and does not throw out any of the optimal solution is formally explained in the Appendix).

**Figure 4:**
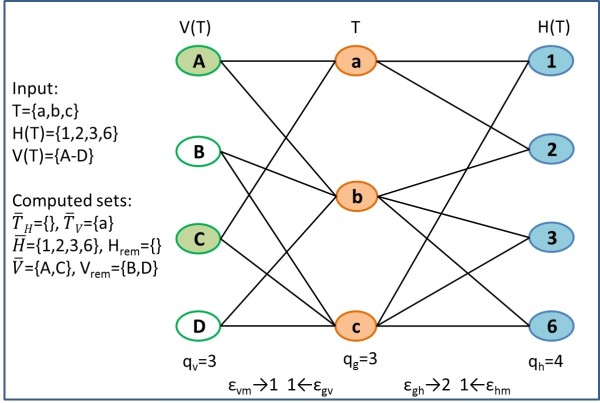
**Example of generating maximal quasi-modules for the path *T***_**1**_** = {*c*,*b*,*a*} from Figure **3**(c).** The algorithm first identifies the genes that are not satisfied with the miRNA sets $\bar{T_{h}}=\{\}$ and $\bar{T_{v}}=\{a\}$ (*a* is the only gene which is connected by an edge to just 2 out of 4 viral miRNAs). Next the miRNAs which are connected to all the genes in sets $\bar{T_{h}}$ and $\bar{T_{v}}$ are identified as $\bar{H}=\{1,2,3,6\}$ and $\bar{V}=\{A,C\}$ (shaded circles). Then these sets are extended by enumerating the remaining miRNAs (white circles). This enumeration yields two quasi-modules which appear in Figure 2**(a)** and **(b)**.

**Stage 2: Traversing the enumeration tree for module extraction.** After the tree is fully developed, its leaves are traversed for identifying paths (ending in these leaves) that meet the quorum criteria on the number of genes (*q*_*g*_). Then the subgraph induced by the genes and the miRNAs associated with each leaf are further processed to identify legitimate modules (maximal with respect to containment) within this subgraph (Figure 4).

Upon completion, the algorithm outputs a list of the *maximal quasi-modules* that satisfy the quorums and error constraints (Figure 2). Next, the p-values of these modules are computed as described below. The modules are then filtered and reported according to ascending p-value.

Note that in order to apply strong pruning in early stages of the enumeration, the insertion into the tree is sorted by increasing order of the number of human miRNAs targeting each gene.

#### Statistical significance of modules - assigning a p-value to a module

Since we search for modules in specific categories of interest, we assess the statistical significance of each quasi-module <*H*,*V*,*T*> in a category *C*, by a sampling procedure as follows. We sample from the full set of genes a subset of |*C*| genes with the distribution of the gene lengths (in our case, the genes^′^3^′^UTRs) as in *C*. From the sampled set of genes we find the maximal set of target genes *T*^′^that can form a quasi-module with the miRNA sets *H* and *V* under the quorum and error constraints. We perform this sampling procedure 10,000 times, and store the distribution of the sizes of *T*^′^. We report for each module the probability that the size of *T*^′^is at least as the size of *T*. This probability is our p-value.

#### The parameters for the quasi bi-targeting algorithm

We used 207 KEGG pathways that contain 10-300 genes from our dataset. For each pathway, we built a bi-partite graph as described in the methods section, where the human and viral miRNAs are listed in Tables 1 and 2. The gene set consisted only of genes that belong to the explored pathway. The edges of the graph were determined by the target prediction results. We tested five sets of parameters on the 207 KEGG pathways. The parameter sets are summarized in Table 3. In the first set we started with minimal quorum constraints for the number of genes, and human and viral miRNAs, and no errors were allowed, similarly to the strategy we employed in [15]. 

**Table 3:** Different sets of parameters for the module search algorithm

**Set No.**	**Parameters**						
	***q***_***h***_	***q***_***v***_	***q***_***g***_	***ϵ***_***hm***_	***ϵ***_***vm***_	***ϵ***_***gh***_	***ϵ***_***gv***_
1	2	2	3	0	0	0	0
2	2	2	3	0.1	0.1	0.1	0.1
3	3	2	4	0.1	0.1	0.1	0.1
4	3	2	4	0.2	0.2	0.1	0.1
5	3	2	5	0.2	0.2	0.1	0.1

The table displays quorum parameters for human and viral miRNAs and human genes (columns 2-4) and ranges of error thresholds (columns 5-8). For error threshold *a.b*we executed the enumeration process with error threshold *a* and if no modules were found, we increased the thresholds one by one, and ran the process again. We stopped raising the error thresholds if the enumeration process resulted with at least one significant module (p-value<0.0001) or when the errors reached the maximal value *b*.

In order to compare our new flexible method with the previous version of bi-targeting [15], we raised the error thresholds from zero to one (denoted as 0.1 in Table 3) - in the second set. In our choice of error parameters, we aimed to allow the minimal error which still yields significant modules. Therefore, when running the enumeration algorithm with this set of parameters, we started by setting the allowed error thresholds to zero, and if no modules were found, we ran the process again by increasing each of the error thresholds alternately. We stopped raising the errors if the enumeration process resulted with at least one significant module (p-value<0.0001) or when the error thresholds reached the maximal value (one in this case).

In the third set (Table 3) we raised the quorum on the number of human miRNAs and genes, and allowed all errors to reach a threshold of one, while in the fourth set the thresholds *ϵ*_*hm*_ and *ϵ*_*vm*_ were raised to two. Finally in set 5, we raised the quorum threshold on the number of genes to be five.

## Results

Human cytomegalovirus (HCMV or HHV5) belongs to the beta subfamily of herpesviridae. Following primary infection, the virus establishes life-long latent infection with episodes of reactivation, mainly in the immune compromised host. HCMV employs diverse mechanisms for the regulation of the host system in ways that are advantageous to the virus [35]. HCMV, as other members of the herpes family, encodes for miRNAs, which were shown to participate in the complex regulation of host cell metabolism and to assist in establishing latency and immune evasion [36]. Several viral and host genes were shown to be targeted by HCMV miRNAs, for a review see [36]. We applied our system to the discovery of quasi-modules of HCMV and human miRNAs and their target human genes, where the human miRNAs are down-regulated upon infection.

### The target prediction results

We supply in Tables 1 and 2 the number of predicted target genes for each miRNA in the dataset. In these results a gene is considered to be a target of a miRNA if the miRNA is predicted to target at least one of the mRNA transcripts of the gene (for some genes in our dataset there are several 3’ UTR transcripts).

### Results of the bi-targeting algorithm

In Table 4 we present, for each set of parameters, information on the number of pathways for which modules were found, and the total and average number of found modules. For each pathway, we counted how many modules were found by the enumeration algorithm and how many of them were statistically significant (p-value<0.0001). We noticed in our results, that the introduction of error flexibility into the module search, results in modules with high overlap in genes or miRNAs. Therefore, we denoted a module as a *redundant module* if there was another module in the same pathway which was identical to it in its gene and miRNA content, except for a variation of up to one gene or miRNA. We counted how many modules among the significant ones were non-redundant. In our results, a pathway is considered to have modules, only if the enumeration process yielded at least one significant module. In the second column of Table 4, we show how many pathways had modules in each of the five parameter sets. For this number of pathways, we show the total number of modules found by the enumeration algorithm, the number of significant modules, and the number of non-redundant modules (see columns 3-5). In column 6, the average number of non-redundant modules among these pathways is shown.

**Table 4:** **Results of the module search algorithm on the parameter sets found in Table **3

**Set No.**	**No. of KEGG**	**No. of modules**			**Average No.**
	**pathways with**	**All**^***b***^	**Significant**^***c***^	**Non-redundant**^***d***^	**of modules**
	**modules **^***a***^				**per pathway**^***e***^
1	3	5	5	5	1.66
2	72	957	375	278	3.86
3	25	290	162	122	4.88
4	69	934	434	306	4.43
5	45	514	202	143	3.17

The parameter sets 1-5 are listed in Table 3 and described in the Methods section. ^*a*^The number of pathways for which significant modules (p-value<0.0001) were found; ^*b*^The number of modules found by the enumeration algorithm in the pathways counted in ^*a*^; ^*c*^The number of modules with p-value<0.0001 among the modules in ^*b*^; ^*d*^The number of non-redundant modules among the modules in *c*. A module is considered to be redundant, if there is another module in the same pathways which is identical to it in gene and miRNA content, except for a variation of up to one gene or one miRNA; ^*e*^The average number of non-redundant modules for the pathways in ^*a*^. Note that set number 1 corresponds to the results of running the algorithm of [15] on the datasets in this paper.

#### Comparing the results of different parameter sets

In the first set we started with quorum thresholds of *q*_*h*_=*q*_*v*_=2 and *q*_*g*_=3 and all the errors were set to 0, which yielded only 5 significant modules among 3 pathways. Note that this setting corresponds to running our previous algorithm [15], which computes full cliques and no errors are allowed. When we increased the allowed errors to 1, the number of modules and the number of pathways dramatically increased (set 2). This reflects the importance of error flexibility which we have added in the quasi bi-targeting algorithm. The transition from set 3 to set 4, in which we increased two of the allowed error thresholds from 1 to 2, also resulted in more modules and additional pathways with modules. The transitions from the second set to the third, and from the fourth set to the fifth, where only the quorum threshold were increased without changing the error thresholds, decreased the number of modules and the number of pathways as expected.

#### The distribution of the pathways with modules among KEGG categories

**Figure 5:**
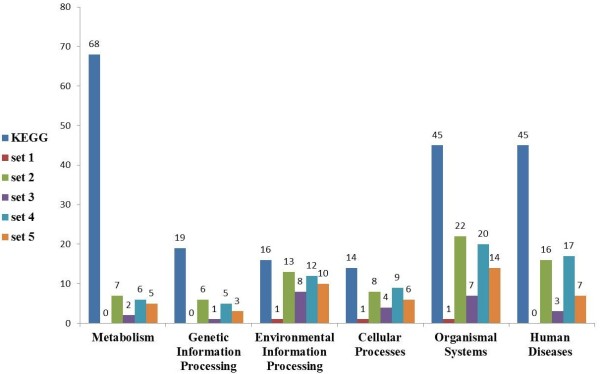
**The distribution of KEGG pathways with modules among the main KEGG categories for parameter sets 1-5.** The leftmost column in each category indicates the number of pathways containing 10-300 genes in the KEGG database [34], and columns 2-6 correspond to the resulting number of pathways for parameter sets 1-5, respectively.

We divided the KEGG pathways in our study into six categories according to the KEGG database [34]: metabolism, genetic information processing, environmental information processing, cellular processes, organismal systems and human diseases. In Figure 5 we show the distribution of the pathways with modules from Table 4 in these KEGG categories. The two categories of metabolism and genetic information processing, contain only few pathways with modules on which we do not elaborate here. Among the 16 pathways belonging to the environmental information processing category, 13,8,12 and 10 of them contain significant modules in sets 2-5, respectively. This category includes sub-categories like signal transduction. In the cellular processes category, which contains 14 pathways, 8 (set 2) and 9 (set 4) pathways contain modules, mainly in cell communication and cell growth and death sub categories. The organismal systems category which consists of 45 pathways, contains modules in 22 (set 2), 20 (set 4) and 14 (set 5) pathways, where 7, 7 and 6 of them respectively, belong to the immune system sub-category (consisting of 15 pathways). As for the 45 pathways in the human diseases category, 16 (set 2) and 17 (set 4) of them contained modules, where most of the pathways belong to the sub-categories of cancers and infectious diseases. 

We observe that the categories and the sub-categories that had many pathways with modules are known to be affected by viral miRNAs. For example, EBV miR-BART targets the host pro-apoptotic gene PUMA [37]. HCMV miR-UL112 modulates host immune responses by targeting the host gene MICB [5]. Some recent high-throughput studies showed that viral miRNAs target mRNAs with roles in the innate immunity, stress response, cell signaling, transcription and apoptosis [16,38].

#### The predicted modules and supporting evidence

To establish an infection, viruses need to suppress the innate and the acquired host immune responses. The gate keepers include antiviral activity induced by IFNs, the chemotaxis of immune cells induced by chemokines and cytokines and activation of NK cells. Thus, we chose to analyze in more detail two modules that were found by the enumeration process from two different pathways from the immune system sub-category. The first module was found in the results of the fourth parameter set and the second one in the results of the fifth parameter set.

The full set of potential modules, including the information on miRNA-mRNA interactions in each module, can be obtained at (http://www.cs.bgu.ac.il/∼vaksler/QuasiBiTargeting.html). To validate these modules we supply a comprehensive overview, based on literature, of the miRNAs and the genes participating in the module. We concentrate on the antiviral activity of the genes and on the mechanisms that viruses apply to suppress these genes. Finding evidence for the above activities may support our predictions and suggests that miRNAs are an additional route in the viral strategy to manipulate the process carried out by the host.

**Figure 6:**
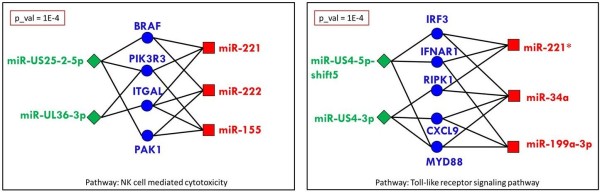
**Two predicted modules.** Two of the modules that are found by our method.

**Figure 7:**
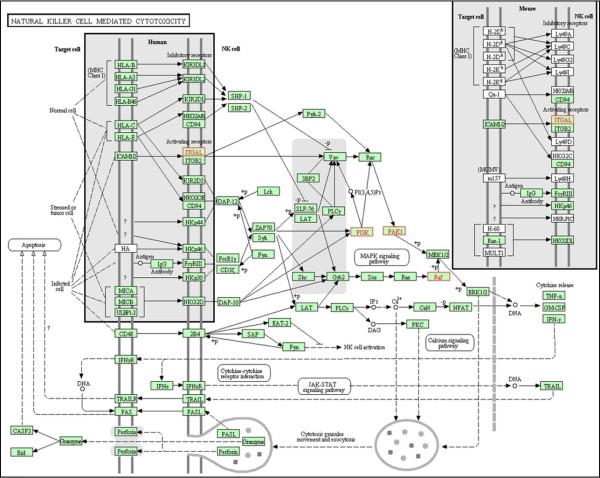
**Natural killer cell mediated cytotoxicity pathway figure supplied by KEGG [**[34]**].** The genes of module 1 reported in this study (see Results section in the main text) are in red.

**Module 1** consists of four genes from the “natural killer cell mediated cytotoxicity” pathway, three human miRNAs and two viral miRNAs (see Figure 6 and Figure 7). Natural killer (NK) cells are cytotoxic cells of the innate immune response that play an important role in eliminating virus-infected cells in early stages of the infection. NK cells are important for controlling CMV infections both in mice and in humans [39]. Furthermore, CMV encodes numerous proteins that interfere with NK cell activity [40]. All genes found in this module, are candidates to be down-regulated by HCMV, supported by the following information. 

The gene **ITGAL** (CD11a) mediates adhesive cell-cell-interactions, by binding to its ligand ICAM-1. Both ITGAL and ICAM-1 were shown to have important roles in NK cell-mediated cytolysis [41]. In addition, Ito et al. [42] showed that these genes are involved in NK-cell mediated DNA-fragmentation of CMV-infected cells. **PAK1** was not yet reported in the context of CMV infection, but it was shown that PAK1 plays an important role in activating antiviral signaling pathways in HCV infection [43,44]. Both genes have proven antiviral activity, which makes them candidates for down-regulation by CMV. Two additional genes in this module are **PIK3R3** and **BRAF**. A study by Challacombe et al. [45], which measured the human mRNA expressions after HCMV infection at different time points, reported that both PIK3R3 and BRAF expressions were increased in the first hour after HCMV infection, and their expression started to decrease 24 hours post infection. This pattern of expression may match the explored mode of regulation in the following manner:(i) the genes were initially up-regulated probably as a response of the host to the infection; (ii) 24 hours post infection, the HCMV miRNAs which are already expressed [46], could target these genes, and thus cause their down-regulation.

As for the human miRNAs found in this module, some of their functions have been studied before. **miR-221** and **miR-222** are encoded in tandem on chromosome X [1], and their targets were extensively studied [47]. For example, they were discovered to induce cell growth and cell cycle progression via direct targeting of p27 and p57 in various human malignancies [48,49]. Interestingly, these miRNAs were shown to target also ICAM-1 [50,51], which is the ligand of ITGAL gene, found in our module.

**miR-155** is located within the B-cell integration cluster (BIC) on chromosome 21, and is involved in cancer and other biological processes such as inflammation, immunity and haematopoiesis (see review by Faraoni et al. [52]). Interestingly, both KSHV and Marek’s disease virus (MDV-1) (both oncogenic viruses) encode miR-155 orthologs [17,53]. In addition EBV up-regulates miR-155 production in infected B cells [54]. It therefore seems that down-regulation of specific host genes by either miR-155 itself, or by viral orthologues of miR-155, might facilitate the replication of a range of different herpesviruses [55]. None of the known HCMV miRNAs shows homology to miR-155. Thus, our results suggest that following the down-regulation of miR-155 by the host, the virus compensates for part of its functions by its own miRNAs, which are found in this module, miR-US25-2-5p and miR-UL36-3p. **miR-US25-2-5p** was recently shown to reduce viral replication and DNA synthesis of HCMV and other DNA viruses (such as HSV-1 and adenovirus) [56], suggesting that this miRNA targets host genes that are essential for viral growth. In our module this miRNA has a different role by targeting genes that lead to antiviral response. No viral and host targets were reported for **miR-UL36-3p** yet.

**Figure 8:**
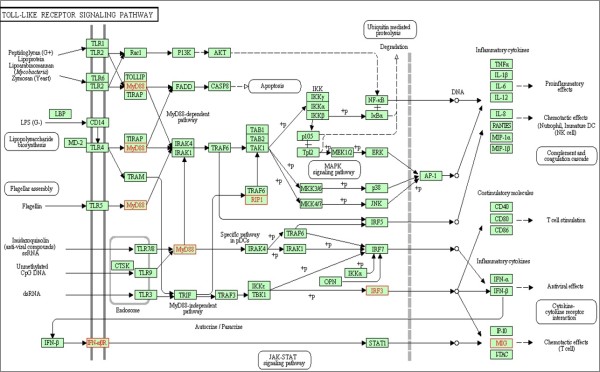
**Toll-like receptor signaling pathway figure supplied by KEGG [**[34]**].** The genes of module 2 reported in this study (see Results section in the main text) are in red.

**Module 2** consists of five genes that participate in the “Toll-like receptor signaling pathway”, three human miRNAs and two HCMV miRNAs (Figure 6 and Figure 8). Toll-like receptors (TLRs) play a critical role in host defense by sensing invading pathogens and initiating innate and adaptive immune responses. TLR signaling proceeds via two downstream pathways: the MyD88-mediated pathway, and the TRIF-mediated pathway [57] (see Figure 8). The former causes activation of the transcription factor NF-kB, which activates various genes contributing inflammatory reactions. The latter causes induction of IFNs, whose stimulation leads cells to antiviral state. In what follows we describe the known facts that link the genes in the module to viral infections. 

The gene **IRF3** regulates the expression of Type I IFNs (cytokines with antiviral activity), and thus controlls viral infection and virus replication. Several viruses have evolved mechanisms through which they can circumvent the activation of IRF3 and block innate responses, including BHV1 [58], HHV6 [59], KSHV [60] and EBV [61]. In addition, HCMV encodes a protein pp65, that subverts the activation of IRF3 by inhibiting its nuclear accumulation [62]. **RIPK1** (RIP1) is a cellular kinase which has a central role in several biological pathways [63], a fact that makes it an ideal target for inhibition by a virus. It was shown that Mouse cytomegalovirus (MCMV) encodes a protein M45, which binds to RIP1 and functions as a viral inhibitor of RIP1-mediated signaling. The UL45 protein of HCMV shares sequence homology with M45; deletion of UL45 results in only minor defects in viral replication *in vitro*[64]. Thus, it is reasonable to assume that miRNAs can be the regulators of RIP1.

The gene **IFNAR1** encodes a membrane protein that forms one of the two chains of a receptor for interferons alpha and beta that play a major role in host defenses against the viruses. Several viruses were shown to lead to a decrease in IFNAR1 expression levels, including West Nile virus (WNV) [65], vesicular stomatitis virus (VSV) and hepatitis C virus (HCV) [66].

In addition, the **MyD88** gene has been shown to be important in host defense to a number of viruses including Lymphocytic choriomeningitis virus (LCMV) [67], MCMV [68], and HSV [69]. As for the **CXCL9** (MIG), Salazar-Mather and colleagues have demonstrated that this gene is important in viral clearance in mice infected with MCMV [70,71].

The results of our algorithm yield combinations of viral and human miRNAs that target the genes above. Members of the miR-34 family, including **miR-34a**, have verified target genes with functions in the cell-cycle control and the DNA damage response [72]. **miR-199a-3p** was shown to down-regulate components of the PI3K/Akt/mTOR pathway, as well as other pathways relevant to HCMV biology [32]. No validated targets are reported for miR-221*. Both viral miRNAs found in this module were recently identified using Deep Sequencing [29,30], and their functions were not yet investigated.

The two modules presented above contain genes that have a strong antiviral activity and a tight connection to the function of HCMV. Thus, upon infection, the host is motivated to up regulate them while the virus has much to gain by achieving the opposite effect. Our results suggest that this interplay may be achieved by the combined action of human and viral miRNAs, where the human miRNAs are down-regulated and the viral miRNAs are expressed to replace their activity.

## Discussion

miRNAs are key regulators of many biological processes produced by both viruses and their hosts. Although the functions of the majority of viral miRNAs are currently unknown, it is suggested that miRNAs greatly contribute to host-viral interactions [73]. Identification and validation of miRNA targets remains a hard problem, because there is large number of potential targets for each miRNA. In addition, mRNAs can be targeted by multiple miRNAs. These potential interactions create a complex network of miRNAs and mRNAs. Focusing on small sets of miRNAs and their effects on particular biological pathways may give a significant advantage in target identification.

In this work we focus on finding modules of viral and human miRNAs and their common target genes in specific biological processes. In our modules, the viral miRNAs follow the compensating mode of regulation with the human miRNAs. In this mode of regulation, human miRNAs are down-regulated upon infection, probably by the host’s machinery, in order to up-regulate antiviral genes. To compensate for this down-regulation, viral miRNAs are expressed to target the antiviral genes. Finding modules is achieved by applying our bi-targeting algorithm and integrating three sources of information: target prediction, a new expression data (supplied in this study) of human miRNAs in infected vs. uninfected cells, and annotation of the biological pathways (KEGG). We treat all the down-regulated host miRNAs as if they were down-regulated by the infected host, regardless of the factors that cause it. This is an assumption we make, even though it can not be inferred solely from expression data [20]. Among the modules found by our method, we report those that were statistically significant, as computed by the sampling procedure that we describe in the Methods Section.

Our bi-targeting algorithm is related to bi-clustering methods applied on bipartite graphs [27]. In our model the graph is a two-sided bi-partite graph, and the goal is to find two sided bi-cliques. In our previous work [15], the output of the bi-targeting algorithm was complete bi-cliques. This resulted in very few modules that were very small (consisting of 2-3 genes, and 1-2 miRNAs).

To add flexibility to the modules, we relax in this paper the bi-targeting algorithm to compute coherent sub-graphs which are not necessarily complete bi-cliques. This relaxation yields quasi-modules, where many more interactions are captured and reported. Our results indicate that such quasi-modules more significantly capture miRNA-target interaction signals. The disadvantage of the method, is that additional parameters (error thresholds) have to be introduced to the search algorithm.

We applied our method to study the miRNA compensating effects in HCMV infection. The complex lytic and latent phases of HCMV are dependent on its ability to regulate many aspects of host immune responses and cell biology. Learning the biological roles of miRNAs during HCMV infection may pave the way to finding a novel class of therapeutic targets for this virus.

We describe in detail two of the found quasi-modules, and supply supporting evidence from the literature. Genes in these modules, were previously shown to have an antiviral activity (see references in the Results section). Thus the host is motivated to up-regulate these genes, by e.g., down-regulating its miRNAs that target them, and the virus is motivated to express its own miRNAs to achieve the opposite effect.

We would like to note that although in this study we used our quasi bi-targeting algorithm to find modules in the compensating mode, it could be used to find modules in other modes of mutual regulation by host and viral miRNAs.

## Appendix

### Quasi-Bi-Targeting (QBT) enumeration algorithm

#### Formal details of the algorithm

Denote by *T* the set of genes in a path from the root of the tree to a leaf, and by *H*(*T*) and *V*(*T*), the lists of human and viral miRNAs stored in the leaf. Denote by *T*(*mir*), the genes that belong to *T* and are targeted by miRNA *mir*. We denote by *H*(*gene*) and *V*(*gene*), the human and viral miRNAs that belong to *H*(*T*) and *V*(*T*) respectively, and target *gene*. Let *ϵ*_*hm*_, *ϵ*_*vm*_, *ϵ*_*gh*_ and *ϵ*_*gv*_ denote error tolerance thresholds.

In stage 1, for each newly generated path in the tree, the sets *H*(*T*) and *V*(*T*) are computed, such that these miRNAs target at least |*T*|−*ϵ*_*hm*_and |*T*|−*ϵ*_*vm*_genes in *T*, respectively. The following observation asserts that the size of the miRNA sets *H*(*T*) and *V*(*T*) is monotonically non increasing with the extension of the gene set along a developing path.

##### Observation 1

***The anti-monotonicity property ofH***(***T***)***andV***(***T***)**.*For every supersetT***^***′***^***of T,H***(***T***^***′***^)**⊆*H***(***T***)***andV***(***T***^***′***^)**⊆*V***(***T***),***thus*|*H***(***T***^**′**^)**|≤|*H***(***T***)**|**and **|*V***(***T***^***′***^)**|≤|*V***(***T***)**|*.***

##### Proof

By definition of *H*(*T*), ∀*mir*∈*H*(*T*^′^) it holds that |*T*^′^|−|*T*^′^(*mir*)|≤*ϵ*_*hm*_. In addition, |*T*|−|*T*(*mir*)|≤|*T*^′^|−|*T*^′^(*mir*)|. Thus |*T*|−|*T*(*mir*)|≤*ϵ*_*hm*_, which means that *mir*∈*H*(*T*), and *H*(*T*^′^)⊆*H*(*T*). The same holds for *V*(*T*). □

Definition 1 (in Methods) leads to the following claims regarding the sought modules.

##### Claim 1

For every *gene*∈*T*, |*H*(*gene*)|≥|*H*|−*ϵ*_*gh*_≥*q*_*h*_−*ϵ*_*gh*_and |*V*(*gene*)|≥|*V*|−*ϵ*_*gv*_≥*q*_*v*_−*ϵ*_*gv*_. □

##### Claim 2

For every pair of genes *gene*1,*gene*2∈*T*, |*H*(*gene*1)∩*H*(*gene*2)|≥*q*_*h*_−2∗*ϵ*_*gh*_and |*V*(*gene*1)∩*V*(*gene*2)|≥*q*_*v*_−2∗*ϵ*_*gv*_. □

##### Proof

|*H*(*gene*)|≥|*H*|−*ϵ*_*gh*_and |*V*(*gene*)|≥|*V*|−*ϵ*_*gv*_. Therefore, |H(gene1)∩H(gene2)| = $|H(\mathrm{gene}1)|+|H(\mathrm{gene}2)|-|H(\mathrm{gene}1)\cup H(\mathrm{gene}2)|\geq |H(\mathrm{gene}1)|+|H(\mathrm{gene}2)|-|H|\geq 2(|H|-\epsilon_{\mathrm{gh}})-|H|=|H|-2*\epsilon_{\mathrm{gh}}\geq q_{h}-2*\epsilon_{\mathrm{gh}}$. The same holds for viral miRNAs (*V*). □

The following conclusions apply Observation 1 and Claims 1 and 2 to prune the enumeration tree.

##### Conclusion 1

If *H*(*T*)<*q*_*h*_, then for every superset *T*^′^of *T*, *H*(*T*^′^)<*q*_*h*_. The same holds for *V*(*T*). During the construction of the tree, if for a certain path *T*, *H*(*T*)<*q*_*h*_or *V*(*T*)<*q*_*v*_there is no need to extend this set further, and the path *T* is pruned from the tree. □

##### Conclusion 2

If there exist a *gene*∈*T*, for which *H*(*gene*)<*q*_*h*_−*ϵ*_*gh*_or *V*(*gene*)<*q*_*v*_−*ϵ*_*gv*_; or a pair of genes *gene*1,*gene*2∈*T*for which |*H*(*gene*1)∩*H*(*gene*2)|<*q*_*h*_−2∗*ϵ*_*gh*_or |*V*(*gene*1)∩*V*(*gene*2)|<*q*_*v*_−2∗*ϵ*_*gv*_, there is no need to extend *T* further, and this set can be pruned from the tree. □

A path in the tree which survives a pruning based on Conclusions 1 and 2 can be defined as follows:

##### Definition 2

**A surviving path** is a path *T* in the tree, such that the miRNA sets, *H*(*T*) and *V*(*T*), in its leaf fulfill the following conditions: **(1)**∀*mir*∈*H*(*T*),|*T*(*mir*)|≥|*T*|−*ϵ*_*hm*_, **(2)**∀*mir*∈*V*(*T*),|*T*(*mir*)|≥|*T*|−*ϵ*_*vm*_, and **(3)**∀*gene*∈*T*,|*H*(*gene*)|≥*q*_*h*_−*ϵ*_*gh*_and |*V*(*gene*)|≥*q*_*v*_−*ϵ*_*gv*_.

After the tree is fully developed (in stage 1), the surviving paths are traversed to identify paths which contain at least *q*_*g*_ genes (stage 2). Note that Definition 1 (quasi-module) deviates from Definition 2 (surviving path). In the latter, the error allowed for each gene is in respect to the miRNA quorum, while in the former the error is in respect to the size of the final module.

Therefore, it is possible that some of the genes in a surviving path *T* do not satisfy the error constraint with respect to the full sets *H*(*T*) or *V*(*T*), i.e., there exists a *gene*∈*T* such that |*H*(*gene*)|<|*H*(*T*)|−*ϵ*_*gh*_ or |*V*(*gene*)|<|*V*(*T*)|−*ϵ*_*gv*_. In this case, we search for maximal subsets of *H*(*T*) and *V*(*T*) that can form quasi-modules with *T*.

##### Generating maximal quasi-modules

The generation of these maximal subsets is done by first identifying the genes in *T* that do not satisfy the constraints with *H*(*T*) and *V*(*T*), denoted as $\bar{T_{h}}$ and $\bar{T_{v}}$ respectively. If the sets are empty, a quasi-module <*H*(*T*),*V*(*T*),*T*> is added to the output.

Otherwise, we identify two subsets of human and viral miRNAs $\bar{H}$ and $\bar{V}$ (*core sets*), that are connected to all the genes in $\bar{T_{h}}$ and $\bar{T_{v}}$ respectively. These core sets will be included in all the maximal quasi-modules such that *T* is their target set.

Next, we enumerate the remaining sets of miRNAs $H_{\mathrm{rem}}=\{H(T)\setminus \bar{H}\}$ and $V_{\mathrm{rem}}=\{V(T)\setminus \bar{V}\}$ to find maximal subsets of them, that can form, together with the core sets, quasi-modules with the genes in *T*. The output of this step is all the legal (in terms of quorum and error thresholds) quasi-modules of the form <*H*,*V*,*T*> such that *H*⊆*H*(*T*) and *V*⊆*V*(*T*).

#### Example

We illustrate the algorithm in the following example accompanied by Figures 1, 2, 3, 4. Figure 1 depicts the input graph and the quorum and error constraints. The order of gene insertion is *a*,*e*,*b*,*d*,*c*, due to sorting by the number of their human targeting miRNAs. In Figure 3(a) we show the enumeration tree after inserting the genes *a*,*e*. When we inserted the gene *e* it became the root of a copy of all its preceding siblings: the dummy leaf node and node *a*. Next, the sets of miRNAs in the leaves of the new subtree are updated by eliminating from the copied set, miRNAs that no longer comply with the gene set after its extension with the new gene *e*. For example, in the path *T*={*e*,*a*} there are two genes and each human/viral miRNA should target at least one gene on this path. It follows that the miRNA sets in the leaf of this path are *H*(*T*)={1,2,5,6} and *V*(*T*)={*A*,*B*,*C*,*D*}. Note that human miRNAs 3 and 4 were removed, since both of them are disconnected with more than one gene on the new path (*e*,*a*). Next, the sizes of the miRNA sets are checked for quorum restrictions (|*H*(*T*)|≥4 and |*V*(*T*)|≥3), which are fulfilled in this case. The genes in the path are checked for error satisfaction by the miRNAs in the leaves. For the pair of genes (*e*,*a*), |*V*(*e*)∩*V*(*a*)|=0 which is less than *q*_*v*_−2∗*ϵ*_*gv*_=3−2=1, and therefore this path is pruned from the tree (dotted rectangle).

In Figure 3(b), after the insertion of gene *d*, three paths are pruned due to violation of quorum constraints on either human or viral miRNAs (solid rectangles in the figure). Figure 3(c) illustrates the insertion of gene *c*, and the consequent prunings.

After the full tree is developed, two surviving paths that contain at least *q*_*g*_≥3 genes, *T*_1_={*c*,*b*,*a*} and *T*_2_={*c*,*d*,*b*}, are transfered as input to the procedure that generates maximal quasi-modules with the miRNAs in their leaves.

We demonstrate the application of building maximal quasi modules for the path *T*_1_={*c*,*b*,*a*} in Figure 4. The set of genes *T* cannot form a quasi-module with the full set of viral miRNAs *V*(*T*)={*A*,*B*,*C*,*D*}, because gene *a* is connected just to two out of four of these miRNAs, while the allowed error is one. We therefore search for the maximal subsets of *V*(*T*) that form quasi-modules with *T*. The algorithm first identifies the “problematic” genes with respect to the human and viral miRNA sets: $\bar{T_{h}}=\{\}$, and $\bar{T_{v}}=\{a\}$ respectively. Then miRNAs which target all the “problematic” genes, $\bar{H}=\{1,2,3,6\},\bar{V}=\{A,C\}$, are calculated. Next the procedure extends the module $<\bar{H},\bar{V},T>$ by adding miRNAs from ${\bar{H}}_{\mathrm{rem}}=\{\},{\bar{V}}_{\mathrm{rem}}=\{B,D\}$. This results in two quasi-modules found in Figure 2(a),(b).

The second path which is passed to this procedure is *T*_2_={*c*,*d*,*b*}. Its genes satisfy the error constraint with respect to the full sets of human and viral miRNAs in its leaf (i.e. $|\bar{T_{h}}|=0,|\bar{T_{v}}|=0$), thus a quasi-module consisting, of all the genes and all the miRNAs is added to the output (Figure 2(c)).

## Pseudocode of the enumeration algorithm

### **Algorithm 1:** constructTree

**input:***G* – list of genes from a certain GO/KEGG category. Every gene *x* in this list has a set of human and viral miRNAs targeting it, denoted as *x.h*_*miRs*and *x.v*_*miRs*, respectively. *M*_*H*_, *M*_*V*_ – full list of human and viral miRNAs.**output:** Enumeration tree and a set of quasi-modules *Q*_*M*_ /* Generating the enumeration tree */1 root← new Node();2 dummy ← new Node();3 dummy.*h*_*miRs*←*M*_*H*_;4 dummy.*v*_*miRs*←*M*_*V*_;5 root.children.add(dummy);6 **foreach***gene*∈*G***do**7 node← appendSiblings(*gene*);8 root.children.add(*node*); /* Traversing the enumeration tree */9 **foreach***leaf*∈*root.leaves***do**10 *T*←*leaf.genes*11 *H*(*T*)←*leaf.h*_*miRs*12 *V*(*T*)←*leaf.v*_*miRs*13 **if**|*T*|≥*q*_*g*_**then**14 generateModules(*T*,*H*(*T*),*V*(*T*));

### Algorithm 2: appendSiblings

**input:** gene – a new gene to be inserted into the tree. root – the root node of the enumeration tree. *q*_*g*_,*q*_*h*_,*q*_*v*_– quorum restriction on human genes and human and viral miRNAs in a sought module. *ϵ*_*hm*_,*ϵ*_*vm*_– the error allowed for each human and viral miRNA respectively, with respect to the target genes. *ϵ*_*gh*_,*ϵ*_*gv*_– the error allowed for each gene with respect to the human and viral miRNAs, respectively.**output:** Creates a new node and appends to it (as its children) all its siblings1 node ← new Node(*gene*);2 **foreach***child*∈*root.children***do**3 sibling ← copy(child);4 **foreach***leaf*∈*sibling.leaves***do**5 *T*←*leaf.genes.add*(*gene*);6 *H*(*T*)←*leaf.h*_*miRs*←{*mir*|*mir*∈*leaf.h*_*miRs*∧ targets at least |*T*|−*ϵ*_*hm*_genes };7 *V*(*T*)←*leaf.v*_*miRs*←{*mir*|*mir*∈*leaf.v*_*miRs*∧ targets at least |*T*|−*ϵ*_*vm*_genes };8 **if**|*H*(*T*)|<*q*_*h*_∨|*V*(*T*)|<*q*_*v*_∧**then** /* Prune due to quorum restrictions on miRNA sets */9 Delete the path from the leaf to the first bran- ching node in sibling subtree;10 **if**!*checkConditions*(*T*,*H*(*T*),*q*_*h*_,*ϵ*_*gh*_,*V*(*T*),*q*_*v*_,*ϵ*_*gv*_) **then** /* Prune due to un‐satisfaction ofthe genes on the path by themiRNAs in the leaf */11 Delete the path from the leaf to the first branching node in sibling subtree;12 return node;

### Algorithm 3: checkConditions

**input:***T* – a list of genes.*H*,*V* – lists of human and viral miRNAs.*q*_*h*_,*q*_*v*_ – quorum restriction on human and viral miRNAs.*ϵ*_*gh*_,*ϵ*_*gv*_– the error allowed for each gene with respect to the human and viral miRNAs, respectively.**output:***True* if the conditions are satisfied, *False*otherwise. /* Check condition 1 ‐ Lemma 2 */1 **foreach***gene*∈*T***do**2 **if** ($|H(\mathrm{gene})|<q_{h}-\epsilon_{g_{h}}$) or ($|V(\mathrm{gene})|<q_{v}-\epsilon_{g_{v}}$) **then**3 return *False*; /* Check condition 2 ‐ Lemma 3 */4 **foreach** pair (*gene*_*a*_,*gene*_*b*_) ∈ (*T*×*T*) **do**5 **if** ($|H(\mathrm{gen}e_{a})\cap H(\mathrm{gen}e_{b})|<q_{h}-2*\epsilon_{g_{h}}$) or ($|V(\mathrm{gen}e_{a})\cap V(\mathrm{gen}e_{b})|<q_{v}-2*\epsilon_{g_{v}}$) **then**6 return *False*;7 return *True*;

### Algorithm 4: generateModules

**input***T*,*H*,*V* – lists of genes and human and viral miRNAs.**output***Q* – a set of quasi-modules <*H*^′^,*V*^′^,*T*> such that *H*^′^⊆*H*and *V*^′^⊆*V*.1 $\bar{T_{h}}\leftarrow \emptyset$, $\bar{T_{v}}\leftarrow \emptyset$;2 **foreach***gene*∈*T***do**3 **if** (|*H*(*gene*)|<|*H*|−*ϵ*_*gh*_) **then**4 $\bar{T_{h}}\mathrm{.add}(\mathrm{gene})$;5 **if** (|*V*(*gene*)|<|*V*|−*ϵ*_*gv*_) **then**6 $\bar{T_{v}}\mathrm{.add}(\mathrm{gene})$;7 **if**$|\bar{T_{h}}|=0$ and $|\bar{T_{v}}|=0$**then**8 Q.add(<*H*,*V*,*T*>);9 return;10 $\bar{H}\leftarrow \{\mathrm{mir}|\mathrm{mir}\in H\wedge \mathrm{mir}$ targets all genes in $\bar{T_{h}}\}$11 $H_{\mathrm{rem}}\leftarrow H\setminus \bar{H}$12 $\bar{V}\leftarrow \{\mathrm{mir}|\mathrm{mir}\in V\wedge \mathrm{mir}$ targets all genes in $\bar{T_{v}}\}$13 $V_{\mathrm{rem}}\leftarrow V\setminus \bar{V}$14 *S*← empty stack 15 $\mathrm{S.}\text{push}(T,\bar{H},H_{\mathrm{rem}},\bar{V},V_{\mathrm{rem}})$16 **while** (S is not empty) **do**17 *clique*←*S.pop*();18 *T*←*clique.T*;19 $H\leftarrow \mathrm{clique.}\bar{H}$, *H*_*rem*_←*clique.H*_*rem*_;20 $V\leftarrow \mathrm{clique.}\bar{V}$, *V*_*rem*_←*clique.V*_*rem*_;21 *extended*←*false*; /* we add null to allow extending only human or viral mirs */ 22 **foreach***mirH*∈(*H*_*rem*_∪*null*)**do**23 **foreach***mirV*∈(*V*_*rem*_∪*null*)**do**24 **if***Legal*(*T*,*H*∪*mirH*, *V*∪*mirV*) **then**25 *H*_*rem*_*.remove*(*mirH*) , *H.add*(*mirH*);26 *V*_*rem*_*.remove*(*mirV*) , *V.add*(*mirV*);27 *S.push*(*T*,*H*,*H*_*rem*_,*V*,*V*_*rem*_);28 *extended*←*true*;29 **if**!*extended*∧|*H*|≥*q*_*h*_∧|*V*|≥*q*_*v*_**then**30 *Q.add*(<*H*,*V*,*T*>);

### Algorithm 5: Legal

**input:***T* – a list of genes in the module.*H* – a list of human miRNAs.*V* – a list of viral miRNAs.**output:***True*if all the genes in *T* are satisfied with the miRNA sets *H* and *T*, *False*otherwise.1 **foreach***gene*∈*T***do**2 **if**|*H*(*gene*)|<|*H*|−*ϵ*_*gh*_or |*V*(*gene*)|<|*V*|−*ϵ*_*gv*_**then**3 return *False*;4 return *True*;

## Conclusion

Since not much is known about the function of viral miRNAs, finding modules that link the viral miRNAs and the human miRNAs, under the mode of action proposed by this study, might help in understanding the role of viral miRNAs in the viral combat to survive, on the one hand, and the role of host miRNAs in antiviral responses, on the other hand. Thus the method developed in this work may shed light on these phenomena.

## Competing interests

The authors declare that they have no competing interests.

## Authors’ contributions

EM, ZB and YSA contributed the miRNA expression data. IVL, KK, and MZU developed the algorithms. IVL contributed to code development and data analysis, and found supporting evidence in the literature. All authors drafted the manuscript, and read and approved the final manuscript.

## Supplementary Material

Additional file 1 **Human miRNAs expression data.** An excel file which provides expression of human miRNAs in HFF cells before and after the infection with HCMV. Click here for file
